# Non-Pharmacological Management of Urge Urinary Incontinence in Women between 40 and 65 Years Old: A Systematic Review

**DOI:** 10.3390/nursrep14010015

**Published:** 2024-01-09

**Authors:** Sara Trapani, Giulia Villa, Andrea Poliani, Silvia Gnecchi, Debora Rosa, Duilio F. Manara

**Affiliations:** 1 Department of Obstetrics and Gynecology, IRCCS San Raffaele Hospital, 20132 Milan, Italy; trapani.sara@hsr.it; 2 Department of Biomedicine and Prevention, University of Rome Tor Vergata, 00133 Rome, Italy; 3 Center for Nursing Research and Innovation, Vita-Salute San Raffaele University, 20132 Milan, Italy; poliani.andrea@hsr.it (A.P.); rosa.debora@unisr.it (D.R.); manara.duilio@hsr.it (D.F.M.); 4 Department of Onco-Hematology, IRCCS San Raffaele Hospital, 20132 Milan, Italy; gnecchi.silvia@hsr.it

**Keywords:** urinary incontinence, urgency, urge urinary incontinence, conservative treatments, non-pharmacological interventions, effectiveness, middle-aged women, systematic review

## Abstract

Background: Urinary incontinence (UI) has been identified as a World Health Organization health priority. In particular, urge UI (UUI) refers to urine leakage associated with a sudden and compelling desire to void urine. It affects quality of life more than other kinds of UI, but it is not always treated adequately. For these reasons, this study aimed to evaluate the effectiveness of conservative treatment practices to counteract UUI in women aged 40–65 years old. Methods: This systematic review was conducted following the Joanna Briggs Institute (JBI) methodology. According to the protocol registered in PROSPERO, a systematic search was carried out in the CINAHL, Embase, PubMed, PsycInfo, Scopus and Web of Science databases up to October 2022, to find primary studies meeting the inclusion criteria. Results: Fourteen studies were included. The scientific literature reported different strategies dealing with the problem of UUI, some purely physical, others physical and psycho-educational and others exclusively psychological. Conclusion: Conservative treatments are useful to aid the reduction in UUI episodes in middle-aged women. However, none of them can be considered more effective than others due to the impossibility of conducting meta-analytical analyses. Further studies comparing the effectiveness of conservative treatments for UUI are needed.

## 1. Introduction

Bladder issues and conditions affecting the urinary system, referred to as Lower Urinary Tract Symptoms, are widespread worldwide [1]. One of these problems is urinary incontinence, defined as “the complaint of any involuntary leakage of urine” [2,3]. UI can be classified into three main subtypes. Stress urinary incontinence (SUI) is the urine loss provoked by exertion, physical effort, sneezing or coughing; urge urinary incontinence (UUI) refers to urine leakage accompanied by a sense of urgency that is defined as a sudden and compelling desire to void urine [3,4]; and mixed urinary incontinence consists of the combination of SUI and UUI [2].

Urinary incontinence has been identified as a World Health Organization health priority [5], also because the number of individuals with UI is projected to increase with time [6]. Even though UI is widely discussed in the literature, it remains an undervalued problem [7]. In particular, UUI affects quality of life more than other kinds of urinary incontinence [8], but it is not always treated efficiently [9]; indeed, urgency often is a problem not easily amenable to modification [10]. For this reason, there is a need to plan and evaluate the effectiveness of conservative treatment practices to counteract UUI. 

A worldwide prevalence study conducted in the last decade reported that the number of patients affected by UUI in 2008 was 49 million [6]. Nevertheless, estimates of UUI prevalence differ between studies, so that in the European population, the reported prevalence range is between 1.8 and 30.5%, with greater prevalence among women over 40 years of age [11]. For instance, a study conducted in an Italian urban area [12], investigating UI prevalence by age subgroups in a sample of 2900 women, showed that more than one in four women suffered from incontinence episodes from 40 years of age onwards. In line with statistics, this systematic review focuses on the female population in the range of 40–65 years old, which represents the initiation of the significant occurrence of UI in women [13,14,15,16]. In fact, age represents the first predisposing element to the development of UUI, combined with other risk factors: pre-existing clinical conditions, cognitive disorders and a decline in physical function, e.g., hormonal modifications in menopausal women [17]. 

UUI can make people prone to the manifestation of skin infections, sexual dysfunction and depression, with a consequent impact on women’s quality of life, psychosocial health [10] and economic burden from both a societal and patient perspective [11,13,17]. In particular, a study conducted in the United States [18] estimated that in 2007, average annual per capita costs of UUI were USD 1925:USD 1433 in direct medical (physician visits, anticholinergic medications, physical therapy or surgical procedures), USD 66 in direct non-medical (disposable pads or other hygienic products), and USD 426 in indirect costs (earned salary income). According to the European Association of Urology [19], in 2023, the total economic burden of urinary incontinence in Europe was estimated at nearly EUR 40 billion and a total accumulated economic burden of EUR 320 billion is expected in Europe in 2030 if no action is taken [19]. The costs of assisting people with UI are also significant in terms of time: patients managed in a long-term acute care facility need about 109 min/day for care, corresponding to EUR 31.30 per patient per day [20,21]. Cost of illness studies are useful because they can suggest some potential benefits associated with effective treatments, which is a reason why a resolutive action plan needs to be developed. 

The treatments that currently exist for UUI can be classified into three categories: surgery, pharmacological therapies and conservative interventions. Drug therapies, such as anticholinergic agents (e.g., oxybutynin or tolterodine) targeting the muscarinic receptors in the bladder, represent the mainstay of pharmacotherapy for the treatment of urgency and were described in a study conducted by Tiwari and Naruganahalli in 2006 [22]. The choice of pharmacologic therapy, intravesical Botox injections or alternative therapies such as acupuncture depends on the severity of the problem, on the compliance of the patient and on the side effects of the therapy [10]. Regarding the surgical procedures, the progress in incontinence surgery over the last 25 years has been enormous and it is reassuring for patients that there is a possibility of being cured with these methods. For example, the tension-free mid-urethral sling offers long-term cure rates of 70–90%, but patients with urge incontinence do not benefit at all from surgery and, in any case, when symptoms of urgency are predominant, the first line of treatment should be behavioral modifications or conservative measures such as pelvic floor training [10]. The use of non-pharmacological treatment could represent a significant new strategy to reduce UUI symptoms alone or in combination with drugs therapy. It could allow a reduction in medication intake, representing a valid and effective intervention that actively involves the patient [1]. 

Given the importance of these non-pharmacological methods, exploring the scientific literature is important to understand the types of interventions available and their effectiveness, in order to make informed treatment decisions, based on up-to-date data, and guarantee interventions with a lower impact on side effects and costs compared to pharmacological drugs [1]. 

### Objectives

This systematic review aims to establish the non-pharmacological practices that are effective in managing UUI in women between 40 and 65 years old. 

The specific review questions are:

(I). What types of non-pharmacological practices have been employed to reduce or prevent UUI in women between 40 and 65 years old?
(II). What is the effectiveness of these interventions?

## 2. Materials and Methods

### 2.1. Study Protocol

This systematic review was conducted in accordance with the Joanna Briggs Institute (JBI) methodology for systematic reviews of effectiveness evidence [23]. The review question, outcomes, inclusion criteria and methods of analysis were predefined, and the protocol was registered in the International Prospective Register of Systematic Reviews (PROSPERO), with registration n° CRD42023384195.

### 2.2. Criteria for Considering Studies for This Review

Inclusion and exclusion criteria were outlined according to the population, intervention, outcome measures and study design (PICOS) format [24].

#### 2.2.1. Types of Participants

The current review examined studies that included middle-aged women from various cultural backgrounds and countries, where urinary urgency has been investigated. Women affected by SUI or MUI were excluded from the review. 

Women who had experienced brain disorders or spine injuries were not included due to the potential impact of these conditions on urinary incontinence (UI), which might not align with the experiences of other women facing urgency urinary incontinence (UUI). Additionally, women with cognitive impairments were excluded because their challenges in verbal communication might have hindered them expressing their experiences effectively. Women with urinary incontinence after stroke were excluded because it is common for this specific population to have problems controlling their bladder. This also applies to people admitted to nursing homes (NHs). Finally, hysterectomized women and “new mothers” (until one year postpartum [25]) were not included. This last choice was made due to the frequent transitory condition of UUI in this specific population. 

#### 2.2.2. Types of Interventions

This review evaluated any type of non-pharmacological practice designed to manage UUI in women. 

Individual-focused interventions were included but not limited to: mindfulness-based interventions (e.g., wellness programs), cognitive–behavioral-based interventions, stress reduction practices, lifestyle interventions (e.g., changes to diet and exercise) and educational programs for improving bladder continence. There were no limits to the frequency, intensity and duration of interventions. Studies utilizing multi-faceted interventions were also included. This review considered studies that compared an intervention to another intervention or without a comparator (i.e., no intervention).

#### 2.2.3. Types of Outcome Measures 

This review assessed the efficacy of the described interventions in the treatment of UUI and included, but were not limited to, studies with the following outcome measures: mean incontinence episodes per day, symptom severity, quality of life, psychological symptoms and patient satisfaction.

#### 2.2.4. Types of Studies

This review considered both experimental and quasi-experimental quantitative study designs for inclusion such as: randomized controlled trials, non-randomized controlled trials, before and after studies and interrupted time-series studies. 

Although the protocol of this review also indicated consideration of analytical observational studies including prospective and retrospective cohort studies, case–control studies and analytical cross-sectional studies, no articles were found with these study designs that met the inclusion criteria.

We decided not to include review articles because this kind of study often lacks a specific focus on a particular age range. This could pose challenges in meeting the inclusion criteria related to age and might make it challenging to extract pertinent data relevant to this specific population.

### 2.3. Search Strategies

The JBI suggests a search strategy that combines both electronic and manual searches [23]. A three-step search strategy was used in this review. An initial limited search in the MEDLINE via PubMed database was undertaken using the terms: “urge urinary incontinence”, “non pharmacologic intervention*”, “middle-age women” to enhance search sensitivity and identify further suitable keywords and index terms related to the review question. A systematic search was then carried out in the CINAHL, Embase, MEDLINE via PubMed, PsycInfo, Scopus and Web of Science databases. As the different databases allowed free-term searches, a combination of thesaurus terms and free keywords was applied. Thirdly, the reference list of all identified articles was searched manually for any additional studies. The search was limited to Italian and English language studies. We did not impose any database time filters or other limitations in order to conduct a multi-encompassing research that explored the effectiveness of all possible interventions that could be implemented. The complete search strategy is reported in Supplementary Table S1.

### 2.4. Study Selection

All identified citations were collated and uploaded into Zotero reference manager software [26] and duplicates removed. Study selection was divided in two stages, managed with the help of RAYYAN [27]: (I) titles and abstracts were screened by two independent reviewers (S.T., A.P.) for assessment against the inclusion criteria for the review, and potentially relevant studies were retrieved in full; (II) the full text of selected citations was assessed in detail against the inclusion criteria by two independent reviewers (S.T., S.G.). Any disagreements that arose between the reviewers at each stage of the study selection process were resolved through discussion, or with a third senior reviewer (G.V.). 

### 2.5. Assessment of Methodological Quality

The methodological quality of the selected papers was assessed using the JBI Critical Appraisal Tool for Randomized Controlled Trials (a 13-item checklist) [28] and the JBI Critical Appraisal Tool for Quasi-Experimental Studies (a 9-item checklist) [29]. Three authors (S.T., S.G., A.P.) independently evaluated the methodological quality of the selected studies. Study quality criteria were categorized: Yes, No, Unclear and Not Applicable (NA). Any disagreement between the reviewers was solved through discussion with the fourth senior party (G.V.) when necessary. 

The JBI Critical Appraisal Tool does not define a cutoff for which to consider high- and low-quality studies. In the model of Chaboyer et al. (2020) [30], the quasi-experimental studies that obtained a quality score of at least 60% (6 or more criteria classified as “Yes” out of a total of 9 criteria) were considered of moderate quality, and the randomized controlled trials with a quality score of at least 75% (7 out of 13) were considered of high quality. 

### 2.6. Data Extraction

Data extraction was performed by three independent reviewers using the JBI Data Extraction Form for Experimental Studies [23]. The extracted data included general and specific variables: authors, journal, year of publication, study method, setting, population, sample size, interventions (tools, methods and instruments of training, duration of the course), outcomes and measures, study results, authors’ conclusions, comments and limitations. The described variables were entered into Microsoft Excel (2021 version).

### 2.7. Data Synthesis

Three reviewers contributed to data synthesis, categorizing elements of interest in variable groups. In particular intervention types, healthcare professionals, adverse events and outcomes were synthetized. Concerning intervention types, they were classified in two main categories: non-pharmacological and pharmacological. The first one was divided into physical and psychological interventions. The second category was included as a possible mixed intervention or as a control group. Another content analysis was performed according to the different figures dispensing non-pharmacological and/or pharmacological interventions. For each intervention type, adverse events were identified and described. Finally interventions’ outcomes were categorized in: frequency of incontinence episodes, nocturia, incontinence symptoms, voided volume, patient’s satisfaction, quality of life, distress, discomfort and brain activation. Some visual tools were employed to facilitate an immediate discussion of the data. Graphic representations were descriptive tables in which intervention types, adverse events and outcomes were depicted.

## 3. Results

### 3.1. Description of the Studies

From the database searches, 288 documents were retrieved. For deduplication, all papers were systematically collected and sorted in the Zotero reference manager [26]. After deduplication, 256 articles remained. The selection of titles and abstract reported 68 articles, so 188 documents were considered as not answering the search question. Full texts of the deduplicated articles were searched. 

This study relied upon the University’s library service to interrogate the nursing and biomedical electronic databases. All the documents that were not available were requested by the library service if not found in full text on the electronic databases. Nine reports were not retrieved in full text [31,32,33,34,35,36,37,38,39]. Thus, 59 reports were assessed for eligibility and were analysed in full text. One document was retrieved by consulting the reference lists of the documents included in the database searches [40].

**Figure 1:**
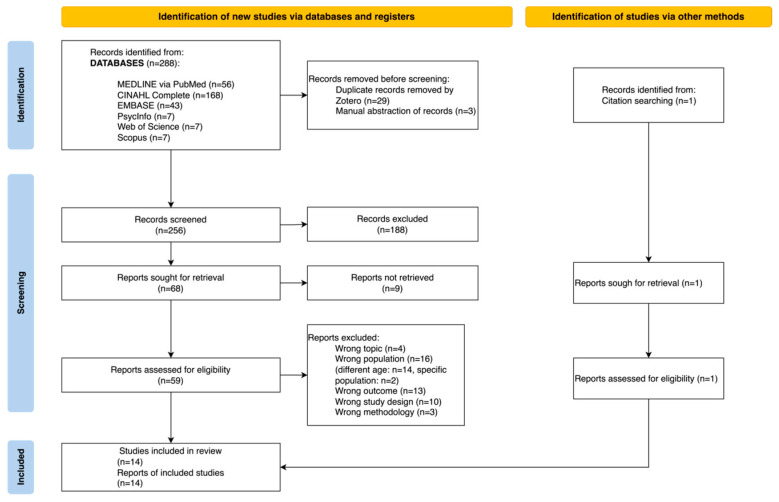
PRISMA Flow Diagram (2020) [41].

The full-text screening eliminated 46 papers because articles had the wrong topic (n = 4), the wrong population (n = 16), the wrong outcome (n = 13), the wrong study design (n = 10) or the wrong methodology (n = 3). After the inclusion of the full texts, 14 studies were included in the systematic review. The entire selection process followed the PRISMA Flow Diagram 2020 [41], as reported below in Figure 1.

### 3.2. Methodological Quality

Results obtained from the JBI Critical Appraisal Tool [23] were reported in two tables according to the study design: quasi-experimental studies and RCTs (Table 1 and Table 2). Among quasi-experimental studies, five articles out of seven [42,43,44,45,46] were classified as moderate quality studies (>60%). Among RCTs, a total of six articles out of seven [47,48,49,50,51,52] were identified as high-quality studies (>75%). In the critical appraisal of quasi-experimental studies, homogeneity was difficult to measure in item numbers 2, 3 and 7. Respectively, the items reached percentages of 57%, 29% and 47% answering “YES”. In addition, item 4 showed that a control group was included in 29% of quasi-experimental studies. In RCTs’ critical appraisal, the table blind mode emerged as being difficult to apply; in fact, item number 4 obtained no positive answers with “YES” (0%) in any study. Moreover, item 6 was difficult to fulfill (57%).

In the model of Todhunter-Brown et al. (2022) [1], the results are reported in different subgroups: physical interventions, physical and psycho-educational interventions and psychological interventions (Table 3).

### 3.3. Physical Interventions

In the literature, various kinds of physical interventions are reported as useful strategies to treat urinary incontinence in women. Tailored exercises such as pelvic floor muscle training and bladder rehabilitation could help women to improve UUI symptoms.

#### 3.3.1. Pelvic Floor Muscle Training and Physiotherapy

Pelvic muscle exercises were proposed for the first time in 1948 by Kegel to reinforce the tone of the periurethral and pelvic floor, through contractions of the perineum muscles [46]. The effectiveness of pelvic floor muscle training in contrasting UUI symptoms was investigated by two studies included in this review [46,52]. 

Wu et al. (2021) [52] randomly assigned their sample to a two-hour behavioral and pelvic floor muscle training (B-PFMT) program or to an informational DVD that proposed a video lasting 20 min. In the B-PFMT group, women were more likely to have fewer urinary urgency episodes at 12 months (cOR = 1.7, 95% CI 1.2–2.3, *p* = 0.002). People in the 20 min video group experienced significantly fewer episodes of urinary urgency at each follow-up time point (at 3 months: cOR = 2.7, 95% CI 2.0–3.8, *p* < 0.001; at 12 months: cOR = 3.5, 95% CI 2.4–4.9, *p* < 0.001; at 24 months: cOR = 3.6, 95% CI 2.6–5.2, *p* < 0.001).

Yoon et al. (2003) [46] compared bladder training versus pelvic floor muscle exercises in the treatment of urinary incontinence in women. On the one hand, the pelvic floor muscle training group was more effective in increasing the peak and the average pressures of pelvic muscle contraction; on the other hand, urinary frequency decreased significantly in the bladder training group during the eight-week treatment period (*p* < 0.01).

Two quasi-experimental studies assessed the effectiveness of physiotherapy for the treatment of UUI and compared this treatment with the pharmacological approach and/or with electric functional stimulation [53,54].

The physiotherapy was defined by Rasero and Mangani as a perineal educational program characterized by three steps: knowledge and information about pelvic physiology, perineal consciousness and Kegel exercises. The same authors defined electric functional stimulation as the usage of an intermittent vaginal electrostimulator with a frequency of 50 Hertz [54].

Pennisi et al. (1994) [53] divided the population sample into three different groups. The first received the physiotherapy treatment alone, the second was treated with oxybutynin 5 mg/3 times a day and the last group received a double treatment composed of physiotherapy and electric functional stimulation. It seems that physiotherapy could be a valid approach to treating UUI. In fact, in the physiotherapy alone treatment group, 83.3% of participants healed and 26.7% improved their condition. Combining physiotherapy and pharmacotherapy with oxybutynin emerged as the most effective treatment with total healing of the participants. Finally, the combination of physiotherapy and electric functional stimulation produced a minor percentage of healed people (62.5%) and a total of 37.5% of women who saw improvements [53].

Rasero and Mangani (2005) [54] conducted a study focusing on the effects of physiotherapy in association with electric functional stimulation, discovering this combination is more effective for women with SUI [54]. 

Differently, the same combination of treatment was not as effective for women who suffered from UUI. In fact, only one woman had improved UUI symptoms, and one reported the complete resolution of their UUI symptoms [54].

#### 3.3.2. Bladder Training

Bladder training, suggested by Jeffcoate and Francis in 1966 [55], is a behavioral approach where the time intervals between voluntary voiding are systematically increased [46]. This kind of intervention was studied by Lagro-Janssen and van Weel (1998) [43], Lauti et al. (2008) [48], Singh and Arya (2011) [44] and Yoon, Song and Ro (2003) [46]. 

In a quasi-experimental study, Singh and Arya (2011) [44] involved twelve women with a history of urge incontinence in a bladder rehabilitation program. The eight-week program included urge suppression techniques and suggestions about fluid manipulation, in order to increase the time interval between each voiding episode. The researchers found statistical differences between pretest and posttest scores regarding the frequency of urination per week (mean difference 3.58, standard deviation 0.669, CI 3.16–4.01; *t* test 18.567, df 11, *p* < 0.0001). After intervention, the frequency of urination per day reduced too (mean difference 2.117, standard deviation 0.3186, CI 1.914–2.319; *t* test 23.013, df 11, *p* < 0.0001), despite it remaining at higher than normal values (from 11.317 to 9.200 voiding episodes/day). Therefore, the study found the bladder rehabilitation program to be a safe and effective conservative intervention to contrast UUI symptoms.

Yoon, Song and Ro (2003) [46] investigated the effectiveness of an 8-week bladder training (group 1) versus pelvic muscle exercises (group 2: 20 min weekly biofeedback session with electromyography for 8 weeks) in the treatment of urinary incontinence in women. The first group achieved better results with a reduction in urinary frequency (frequency of micturition at 8 weeks: 10.4 ± 1.8 n°/day for bladder training group; 14.3 ± 2.4 n°/day for pelvic muscle exercises group; 17.4 ± 1.6 n°/day for control group, *p* < 0.01) compared to the second group.

In their study, Lauti et al. (2008) conducted a comparison of bladder training, drug therapy using oxybutynin at a dosage of 2.5 mg per day and a combination of both approaches. Women allocated to bladder training participated in a one-hour initial session led by a physiotherapist. During this session, a tailored bladder retraining program was established, considering the unique needs of each individual based on a bladder diary. The program encompassed counseling on various aspects including: fundamental anatomy and the typical functionality of the bladder, current bladder-related habits and how they correlate with continence, symptoms related to overactive bladder, identification of triggers causing urgency, fostering healthy bladder practices, and strategies to manage frequency and urgency. Women were educated to try urge suppression when they had urgency, and also with a voluntary pelvic floor muscle contraction. According to adjusted for baseline values, no differences were found between groups in the number of voids per day both at three months (COMBO vs. BRT: −0.1 [−3.5 to 3.3], *p* = 0.971; COMBO vs. DT: 0.8 [−2.6 to 4.2], *p* = 0.630; BRT vs. DT: 0.9 [−2.3 to 4], *p* = 0.576) and at twelve months after intervention (COMBO vs. BRT: 0.2 [−0.8 to 1.3], *p* = 0.668; COMBO vs. DT: 0.7 −0.4 to 1.8], *p* = 0.218; BRT vs. DT: 0.5 [−0.5 to 1.4], *p* = 0.347) [48]. 

In their quasi-experimental study, Lagro-Janssen and van Weel (1998) studied the long-term effects of bladder training for women affected by UUI. During the intervention, women were recommended that voiding should take place at fixed times rather than based on need. A bladder diary was compiled to record the frequency of urine leakages and voidings. The interval between voiding episodes had to be slowly increased by 15 min, to reach a maximum voiding frequency of seven times a day. Compared with the one-year follow-up, the number of continent women after five years remained the same, but the weekly frequency of wet episodes significantly increased between the one- and five-year follow-up, with a mean difference of 0.75 (−1.93–3.43) for women with UUI. Nevertheless, many of the women were satisfied with their treatment. In addition, the study found that only 39% of women continued practicing bladder training once a week or “when necessary” after five years, observing that compliance was an important element for long-term success [43].

Therefore, all the reported studies found that bladder training could represent a possible strategy to contrast UUI episodes and that it is not statistically less valid than other pharmacological and non-pharmacological methods. Nevertheless, the long-term effects of this intervention tend to diminish as time passes and are dependent on the patient’s compliance.

### 3.4. Physical and Psycho-Educational Interventions

Some authors reported both psychological and educational interventions as being effective in UUI reduction. They were often associated with physical exercise or compared to pharmacological treatment. Behavioral training and hypnotherapy appeared to be reasonably investigated in terms of female urinary incontinence treatment.

#### Behavioral Training 

Kafri and colleagues (2006) [42] considered behavioral training to be a rehabilitative practice composed of skills and strategies for preventing incontinence and instructions for daily home practice. 

According to Burgio et al. (2010) [49], behavioral therapy includes: pelvic floor muscle training through vaginal palpation; behavioral strategies to reduce urgency, bladder contractions’ inhibition and prevent incontinence; delayed voiding to increase the intervals between voids for those with a frequency greater than eight times per day; and personalized fluid management for those with excessive urine output (>2100 mL/day). Based on this description, the behavioral programs also encompass lifestyle modifications, as documented by Wu et al. (2021) [52]. A central component of behavioral training is to teach women how to respond adaptively to the sensation of urgency by using urge suppression techniques. The correct behavior should not consist of rushing to the toilet, but instead to pause, to sit down, if possible, to practice relaxing and to contract the pelvic floor muscles several times to suppress urgency, detrusor contraction and urine loss [49]. 

The time and methods for the delivery of the intervention changed according to the studies. Burgio et al. organized four visits in a 10-week period of a combined intervention (open-label, extended-release tolterodine with behavioral training), compared with a pharmacological control group (tolterodine alone) [47,49]. Kafri et al. (2006) [42] proposed five meetings during a 3-month period. Smith, Boileau and Buan (2000) [45] ideated an 8 min educational and motivational video, a journal for education, instructions, and daily documentation forms and home biofeedback. The intervention group in the study of Wadensten et al. (2021) [40] received access to a treatment app (including pelvic floor muscle training, bladder training, psycho-education, lifestyle advice, tailored advice, exercise log, reinforcement messages and reminders).

Regarding the effectiveness of behavioral training in reducing UUI symptoms and the frequency of incontinence episodes, Kafri et al. (2006) [42] found that the rehabilitative group (behavioral training) improved during the 3-month follow-up period, while the pharmacological group (oxybutynin) deteriorated to the mean baseline value (*p* < 0.01) [42]. In another study [40], it was found that incontinence symptoms were lower in the treatment group (behavioral training app) than in the control group (information app) [estimated difference −3.1, 95% CI −4.8 to −1.3]. Incontinence episodes’ frequency reduction from baseline to 15-week follow-up was greater in the treatment group [−10.5, IQR −17.5 to −3.5] than in the information group (*p* < 0.001) [40]. 

Burgio et al. [47] conducted research examining the correlation between behavioral training and drug therapy. Their findings suggested that incorporating behavioral training alongside tolterodine might lead to a reduction in the frequency of incontinence episodes during the active treatment phase (69% combination group vs. 58% tolterodine group; difference, 11 percentage points [CI, 0.3 to 22.1 percentage points]) [47,49]. Nevertheless, the drug and non-pharmacological association does not improve urgency more than the drug alone (*p* = 0.30) nor the ability to discontinue drug therapy and maintain an improvement in urinary incontinence in the long term [47,49]. In addition, combination therapy has a beneficial effect on patient satisfaction, perceived improvement and reduction in other bladder symptoms [47]. Finally, Smith, Boileau and Buan (2000) [45] combined behavioral training with biofeedback and found that women with symptoms of urge incontinence decreased voids per day by a mean of 3.17 for a significant *p*-value < 0.001. Biofeedback is a strategy where a pneumatic sensor measures pelvic muscle contractions and provides immediate feedback, along with software that guides the patient through preset protocols [45].

Despite the heterogeneity of the methods employed to conduct the above searches being high, the studies stated that behavioral training is effective in reducing UUI frequency. 

### 3.5. Psychological Interventions

#### Hypnotherapy

Two RCTs [50,51] investigated the efficacy of hypnotherapy in the treatment of UUI. In both studies, hypnotherapy was considered to be a practice focused on emotions identified by participants associated with UUI. In both studies, eight-weekly, one-hour, one-on-one hypnotherapy sessions were delivered by a certified hypnotherapist for the non-pharmacological group. Meanwhile, the pharmacotherapy group received a drug treatment with oxybutynin 10 mg/day or tolterodine 4 mg/day. In addition, in the study conducted by Komesu et al. (2020) [51], the drug treatment was completed with counseling. The studies did not prove the non-inferiority of hypnotherapy compared to medications; in fact, a reduction in UUI episodes was reported in both treatment groups. Ketai et al. (2021) [50] obtained a change in the UUI episodes’ median of −5 (Q1 −2, Q3 −10) in the hypnotherapy group and of −4.5 (Q1 −3, Q3 −7) in the medication group, with a *p*-value of difference *p* = 0.60, 8 or 12 weeks after the treatment started. Komesu et al. (2020) [51] evaluated the UUI episodes as having a median after 2 months of 1 (Q1 0, Q3 5) in the hypnotherapy group and 1 (Q1 0, Q3 3) for the pharmacotherapy group, after 6 months, the median was 1 (Q1 0, Q3 3) in the hypnotherapy group and 1 (Q1 0, Q3 4) for the pharmacotherapy group, and 12 months after the treatment started, the median was 1 (Q1 0, Q3 3) in the hypnotherapy group and 1 (Q1 0, Q3 5,5) for the pharmacotherapy group. 

In conclusion, hypnotherapy seemed to be an alternative treatment for women with UUI because it demonstrated the same values as the drug therapy in terms of the median change between groups.

### 3.6. Healthcare Professionals Involved and Setting

Healthcare professionals involved in dispensing non-pharmacological interventions were classified into different specialized categories. The main cited figure is represented by physiotherapists with a frequency of 4/14 [47,48,49,53,54], even if their interventions are restricted to the rehabilitation field. The second main professional involved is the nurse (3/14) [46,47,49,54]. Non-pharmacological interventions were also dispensed by hypnotherapists with a frequency of 2/14 [50,51]. Two different groups of healthcare specialists were represented by the same frequency (1/14): occupational therapists and gynecologists [44,52]. In one article, healthcare professionals were identified in general as being responsible for the non-pharmacological intervention. No specific reference was addressed to single healthcare providers [52]. Some articles reported teamwork as a strategic choice to provide the intervention [44,47,49,54]. A total of four articles did not specify the involvement of any healthcare provider [40,42,43,45]; two of these were dispensed in a telematic way [40,45].

### 3.7. Other Outcomes

The included articles considered other outcomes apart from the frequency of incontinence episodes and incontinence symptoms, the main study outcomes. Among secondary outcomes, four studies investigated nocturia [42,46,48,52]. The outcome about quality of life was also considered in four articles [40,42,47,48]. Additional outcomes were singularly cited in one article: voided volume [46], patient’s satisfaction [47], distress [47] and brain activation [50] (Table 4). 

Overall, the studies investigating nocturia found a generally positive effect of the proposed interventions on the number of void episodes during the night, but the superiority of any intervention over others cannot be affirmed. 

Quality of life was investigated as an outcome. Burgio et al. (2008) [47] in their RCT compared combination therapy with behavioral training and tolterodine 4 mg/day versus pharmacological treatment alone [47]. Quality of life was measured using the OABq health-related quality-of-life scale and the Short-Form Health Survey (SF-12). This outcome was discovered to improve in both groups (at ten weeks and eight months postinterventions); however, the differences between groups were small, even if statistical data were not reported by the authors. Kafri et al. (2007) [42] used the Incontinence Quality of Life Instrument (I-QoL). Due to some baseline differences between study groups (rehabilitation group with behavioral training and medication group through oxybutynin 5 mg/day), a repeated-measures analysis was performed. In the within-group comparison, both groups improved significantly over time with respect to urinary symptoms and QoL score (F2,70 = 20.89, *p* < 0.01). A significant negative association was found between the urinary symptoms and the I-QoL at the end of the three-month follow-up period (rp = −0.35 to −0.62, *p* < 0.05). Another study investigated this outcome using the Overactive Bladder Questionnaire (OAB-q) quality-of-life measure. No differences were found between groups in the OAB-q at 12 months (87.9 SD 11.6 bladder retraining, 81.6 SD 19.3 drug therapy and 88.9 SD 9.9 combination) [48]. Finally, Wadensten et al. (2021) [40] assessed quality of life in participants randomized to receive access to a treatment app or an information app, using the ICIQ−Lower Urinary Tract Symptoms Quality of Life Module (ICIQ-LUTSqol). The estimated between-group difference was −6.3 (95% CI −10.5 to −2.1) at the 15-week follow-up [40].

Overall, quality of life improved in every intervention group of the described studies, and when this outcome was measured with different tools. Due to the heterogeneity of the articles, the superiority of any intervention over others cannot be affirmed. 

One study compared bladder training, pelvic floor muscle exercises and a control group without intervention to evaluate the effectiveness for increasing voided volume. It emerged that the bladder training group significantly improved this outcome (*p* < 0.01) compared with the other groups in which no changes were observed [46]. Patient satisfaction and distress were assessed by Burgio et al. (2008) [47] in an RCT comparing combination therapy with behavioral training and tolterodine 4 mg/day versus pharmacological treatment alone. The combination group showed a complete state of satisfaction compared to the pharmacological group (53% vs. 40%; difference, 13 percentage points [CI, 1 to 25 percentage points]) ten weeks after the start of the intervention. Moreover, at eight months, the combination group showed improved outcomes (33% vs. 20%; difference, 13 percentage points [CI, 2 to 24 percentage points]). Distress symptoms were improved in both groups, with better scores in the combination therapy group versus the pharmacological group (*p* < 0.001). In the first ten weeks of the intervention, distress decreased, while in the following period until eight months, the distress scores slightly increased in both groups [47]. Brain activation related to a bladder-filling task was explored in an RCT by Ketai et al. (2021) comparing hypnotherapy intervention with drug intervention (oxybutynin 10 mg/day or tolterodine 4 mg/day). It emerged that there was a reduction in left temporoparietal junction activation (*p* < 0.01) in both groups [50]. 

### 3.8. Adverse Events

In four articles, adverse events were reported in both pharmacological and non-pharmacological interventions [42,47,48,51]. Three of these articles presented the worst adverse events as occurring in the pharmacological intervention group [42,48,51]. Just one article about behavioral training declared the presence of adverse events in a non-pharmacological group [40]. The description of detailed adverse events is reported in Table 5.

## 4. Discussion

The recognition of UI as a significant clinical issue [56] underlines the necessity of identifying effective interventions. The increasing prevalence of UUI has critical implications not only for women’s physical and psychological health [10] but also for their social and economic life [11]. This systematic review aimed to identify the available interventions to contrast UUI, excluding surgery and drug therapy [22]. In this regard, the purpose of this study was relevant for framing up-to-date knowledge in relation to the efficacy of UUI non-pharmacological treatments, closing a current gap in incontinence care, because patients with urge incontinence do not benefit at all from surgery and are often subject to the side effects of drug therapy [10]. 

The literature reports on different strategies to deal with the problem of urinary incontinence, some purely physical (e.g., physiotherapy or bladder training), others physical and psycho-educational (e.g., behavioral training and lifestyle modifications) and others exclusively psychological, such as hypnotherapy. 

The interventions that emerged in this systematic review could support pharmacological therapy or could be used alone to reduce incontinence episodes because their benefits were demonstrated and these results are in line with an overview of reviews published in 2022: pelvic floor muscle training, biofeedback, electrical stimulation and bladder training were found to be more beneficial than a control for curing or improving UI [1]. However, according to our study, the effectiveness of the non-pharmacological interventions cannot be generalized because of the impossibility of conducting meta-analytical analyses due to insufficient statistical data. 

For the same reason, none of the conservative interventions can be considered more effective than the others. Since the evidence does not allow the superiority of an intervention to be supported over others, the involvement of a health professional is necessary to choose the right treatment according to professional experience, resources and patients’ preferences [57].

In order to improve women’s adherence to the therapies, health professionals’ support is essential. The main involved figure is represented by physiotherapists [47,48,49,53,54]: nevertheless, their interventions are restricted to the rehabilitation field. The second main involved leaders are nurses [46,47,49,54], whose preventive, curative, palliative and rehabilitative assistance is of a technical, relational and educational nature [58] and therefore of a broader spectrum. Nevertheless, in order to guarantee constant, continuous and complete care to women with UUI, it is advisable to ensure teamwork and multiprofessional care [44,47,49,54]. Every healthcare professional included in the review, such as hypnotherapists [50,51], occupational therapists and gynecologists [44,52], are able to work on some of the different factors contributing to UUI, and therefore, the collaboration of everyone is necessary [44,47,49,54]. 

Although midwives are competent in counseling women with urogynecological disorders [59], none of the included studies involved this professional in the interventions used to reduce UUI disorders. In fact, midwives have confidence in pelvi-perineal muscles management and are also attentive to the psychological nature of the disease. In addition, they are trained to conduct psycho-educational meetings, mind–body encounters and body awareness to guarantee women’s wellbeing and to prevent UUI complications [60]. 

Health professionals such as physiotherapists and urological nurses with their interventions, act not only on the symptoms of UUI but also on other outcomes, such as increasing satisfaction, reducing distress and improving quality of life [40,42,47,48]. In fact, women who are provided with pharmacological therapies generally receive much less attention than patients treated with conservative treatments who receive additional personal attention [42]. Nonetheless, this study does not allow the identification of the best intervention with respect to these outcomes, and this is also because personal subjectivity is involved [61]. The correlation between objective and subjective outcome measures regarding urinary incontinence is still an open point, and further studies on this topic have been recommended by some authors [46]. Regarding adverse events, this study found major side effects in the case of interventions where a drug therapy was employed [42,47,48,51], demonstrating that non-pharmacological treatments are better in terms of safety. Nevertheless, it is important that non-pharmacological treatments are delivered correctly with the help of a health professional to avoid some adverse events that may still occur, such as inguinal hernia [40] and back pain [51]. 

Scientific evidence reports that physical interventions can be dated, in their origin, to earlier times: for example, bladder training and pelvic floor muscle training date back to the second half of the 1990s [46]. On the other hand, the interest in strategies of a more psychological nature has been discovered in the last years, as demonstrated by the most recent publications on this topic [50,51]. Since the second decade of the 21st century, a change in theories has been taking place. In particular, beyond the use of the most accepted therapies such as pharmacotherapy and physiotherapy, mind–body therapy has emerged as an innovative intervention against UUI. The pioneering part consists of the application of a psychological approach to treat a physical problem. As reported by Komesu et al. (2020) [51], urinary incontinence provokes emotional distress. In fact, it seems that hypnotherapy, promoting brain activation, improves the consciousness and perception of urinary stimuli, reducing the number of incontinence episodes. The effectiveness of treatments is dependent on the period of time dedicated to the interventions, which varies in the included studies. This fact could affect the effectiveness both in the short and long term.

With the passing of time, there is an increasing percentage of incontinence in women after five years of physical intervention such as bladder training. The reduction in the treatment’s effect in the long term seems to be associated with poor compliance and with physiological decline due to aging [43].

### Limitations

This review’s findings have revealed several significant deficiencies in the available literature. In fact, only 14 papers were found. This fact demonstrates that UUI is little explored in the literature even though it creates several problems for people who are affected by it. Thus, the UUI field should be more deeply investigated. 

Most of the included studies presented some limitations such as different follow-up timings. This fact contributes to the heterogeneity of the results and does not allow the interventions to be compared in terms of time. All the included papers showed a small population sample except for the study conducted by Wu and colleagues in 2021 (647 patients). The other population samples varied from 12 participants [44] to 307 patients [47,49]. Different sample sizes do not allow results and data to be generalized to the overall population. Furthermore, most of the included studies did not report sufficient statistical data to warrant statistical and meta-analytical analysis.

In addition, different tools and evaluation scales were employed to measure incontinence and the correlated outcomes. Heterogeneous measures made it difficult to compare studies’ results.

In different studies, such as in Kafri et al. (2006) and Pennisi et al. (1994), the included samples were unblinded to the intervention, so every participant knew the kind of intervention they received [42,53]. When there was an experimental group, the control group also knew the intervention because of the impossibility of blinding the treatments due to their nature.

The language filters used in this review did not allow the inclusion of studies written in other languages different from English and Italian. Thus, there is the possibility that more studies conducted about UUI exist in the literature that could not be included in this review.

Despite lifestyle modifications being included in the psycho-educational interventions, this systematic review has not described the specific behaviours to adopt in depth due to the lack of detailed specification in the included articles.

## 5. Conclusions

This systematic review showed a range of non-pharmacological strategies that can be helpful to reduce or resolve UUI symptoms. Health professionals should include these interventions in the patient pathway assuring the absence of medication-related adverse events. The choice of treatment should be influenced by the patient’s preferences.

The effectiveness of non-pharmacological treatments in patients with UUI is certain but difficult to generalize. They could be safely applied to women with ages ranging from 40 to 65 years, improving their quality of life and reducing symptoms.

### Implications for Research

This systematic review identified a need for more empirical research. There is a need to provide both qualitative and quantitative evidence bases for using non-pharmacological interventions to reduce UUI symptoms (especially using psychological treatments). More studies should include conservative interventions such as yoga and mindfulness that have proven to be effective in the treatment of other chronic diseases. Future research should be expanded to increase the sample sizes of studies, using the same questionnaires and scales to measure symptoms in order to generalize data. Finally, additional studies should specifically focus on lifestyle modifications and the use of psychotherapeutic paths to reduce or prevent UUI.

## Figures and Tables

**Table 1:** JBI Critical Appraisal Tool for Quasi-Experimental Studies [29]. Legend table: Y = YES, N = NO, N/A = NOT APPLICABLE, U = UNCLEAR.

Quasi-Experimental Studies	**Author and Year**	**1**	**2**	**3**	**4**	**5**	**6**	**7**	**8**	**9**	
	Pennisi et al., 1994 [53]	Y	U	N	Y	U	Y	Y	U	N	44%
	Lagro-Janssen and van Weel, 1998 [43]	Y	N/A	Y	N	Y	Y	U	Y	Y	67%
	Smith, Boileau and Buan, 2000 [45]	Y	Y	N/A	N	Y	Y	N/A	Y	Y	67%
	Yoon, Song and Ro, 2003 [46]	Y	U	Y	Y	Y	U	Y	Y	Y	78%
	Rasero and Mangani, 2005 [54]	Y	Y	N/A	N	Y	Y	N/A	U	N	44%
	Kafri et al., 2007 [42]	Y	Y	N	N	Y	Y	Y	Y	Y	78%
	Singh and Arya, 2011 [44]	Y	Y	N/A	N	Y	Y	N/A	Y	Y	67%

(1) Is it clear in the study what is the “cause” and what is the “effect” (i.e., there is no confusion about which variable comes first)? (2) Were the participants included in any comparisons similar? (3) Were the participants included in any comparisons receiving similar treatment/care, other than the exposure or intervention of interest? (4) Was there a control group? (5) Were there multiple measurements of the outcome both pre and post the intervention/exposure? (6) Was follow-up complete and if not, were differences between groups in terms of their follow-up adequately described and analyzed? (7) Were the outcomes of participants included in any comparisons measured in the same way? (8) Were outcomes measured in a reliable way? (9) Was appropriate statistical analysis used?

**Table 2:** JBI Critical Appraisal Tool for Randomized Controlled Trials [28]. Legend table: Y = YES, N = NO, N/A = NOT APPLICABLE, U = UNCLEAR.

Randomized Controlled Trials	**Author and Year**	**1**	**2**	**3**	**4**	**5**	**6**	**7**	**8**	**9**	**10**	**11**	**12**	**13**	
	Burgio et al., 2008 [47]	Y	Y	Y	N	N	Y	Y	Y	Y	Y	Y	Y	Y	89%
	Lauti et al., 2008 [48]	Y	Y	Y	N	N	N	Y	Y	Y	Y	Y	Y	Y	78%
	Burgio et al., 2010 [49]	Y	U	Y	U	U	U	Y	Y	Y	Y	Y	Y	Y	78%
	Komesu et al., 2020 [51]	Y	Y	Y	N	N	Y	Y	Y	Y	Y	Y	Y	Y	89%
	Ketai et al., 2021 [50]	Y	Y	Y	N	N	Y	Y	Y	Y	Y	Y	Y	Y	89%
	Wadensten et al., 2021 [40]	Y	Y	Y	N	N/A	U	Y	Y	Y	Y	U	Y	Y	67%
	Wu et al., 2021 [52]	Y	Y	Y	N	N	Y	Y	Y	Y	Y	Y	Y	Y	89%

(1) Was true randomization used for assignment of participants to treatment groups? (2) Was allocation to treatment groups concealed? (3) Were treatment groups similar at the baseline? (4) Were participants blind to treatment assignment? (5) Were those delivering treatment blind to treatment assignment? (6) Were outcomes assessors blind to treatment assignment? (7) Were treatment groups treated identically other than the intervention of interest? (8) Was follow-up complete and if not, were differences between groups in terms of their follow-up adequately described and analyzed? (9) Were participants analyzed in the groups to which they were randomized? (10) Were outcomes measured in the same way for treatment groups? (11) Were outcomes measured in a reliable way? (12) Was appropriate statistical analysis used? (13) Was the trial design appropriate, and any deviations from the standard RCT design (individual randomization, parallel groups) accounted for in the conduct and analysis of the trial?

**Table 3:** Intervention. Legend table: UI = urinary incontinence; UUI = urge urinary incontinence; MUI = mixed urinary incontinence; SD = Standard Deviation; BRT = bladder retraining; DT = drug therapy; OAB = overactive bladder; FKT = physiotherapy; SEF = Stimolazione Elettrica Funzionale (functional electrical stimulation); BE-DRI = Behavior Enhances Drug Reduction of Incontinence; ↓ = Decrease; ↑ = Increase. “✓” indicates that the item has been included or mentioned in the study.

#	Authors and Year	RCT/ Quasi-Experimental	Intervention 1	Participants	Non-Pharmacological		Pharmacological	Intervention 2	Non-Pharmacological		Pharmacological	Intervention 3	Non-Pharmacological		Pharmacological	Efficacy
					Physical	Psychological			Physical	Psychological			Physical	Psychological		
1	Burgio et al., 2008 [47]	RCT	behavioral training + tolterodine 4 mg/day	A total of 307 women with urge-predominant incontinence randomly assigned in two groups in the study BE-DRI: combination therapy (drug therapy + behavioural training, n = 154, mean age 55.8) and drug therapy alone (n = 153, mean age 58.0).	✓	✓	✓	Tolterodine 4 mg/day			✓					↓ in incontinence episodes was higher in the combination therapy group (69% vs. 58%)
2	Burgio et al., 2010 [49]	RCT	behavioral training + tolterodine 4 mg/day	A total of 307 women with mild and moderate urge and mixed incontinence. Mean age was 58 years old for the drug only group and 55.8 years old for the drug and behaviour group. A total of 153 were assigned to the “drug only” group and 154 to the “drug and behaviour” group. In both groups, treatment was imple- mented in four visits, at intervals of 2–3 weeks, over a period of 10 weeks.	✓	✓	✓	Tolterodine 4 mg/day			✓					↓ in incontinence episodes was higher in the combination therapy group but with women with a mild urge urinary incontinence (*p* < 0.001)
3	Kafri et al., 2007 [42]	Quasi-experimental	behavioral training	A total of 44 women 18 years old or older, mean age: 55 [range 27–69] (mean age: 56.8 with SD = 8 for the medication group, 54.5 with SD = 9.7 for the rehabilitation group). Diagnosis of UUI and OAB in urodynamic testing.	✓	✓		Oxybutynin 5 mg/day			✓					↓ in incontinence episodes was higher in the behavioral training group (*p* = 0.001)
4	Ketai et al., 2021 [50]	RCT	hypnotherapy	A total of 72 women with UUI were recruited from an academic urogynecology clinic and the community at large between March 2013 and April 2016. Hypnotherapy = 36 (mean age 54), Pharmacotherapy = 36 (mean age 57).				Oxybutynin 10 mg/day or tolterodine 4 mg/day			✓					= in incontinence episodes between groups (*p* = 0.001)
5	Komesu et al., 2020 [51]	RCT	hypnotherapy	Women with non-neurogenic UUI for at least three months, Overactive Bladder-Awareness Tool scores ≥ eight, 18 and ≥ three UUI episodes per week. Mean age of the included women: 57.6 (SD = 12.77) hypnotherapy, 59.5 (SD = 10.30) pharmacotherapy.				Oxybutynin 10 mg/day or tolterodine 4 mg/day + counseling			✓					= in incontinence episodes between groups
6	Lagro-Janssen and van Weel, 1998 [43]	Quasi-experimental	bladder training	A total of 88 women between 20 and 65 years (mean age: 50.6, SD = 10.4) presenting UI selected by 13 general practitioners. Type of incontinence: genuine stress: 56 (64%), MUI: 14 (16%), urge incontinence: 18 (21%). Severity of incontinence: severe: 28 (32%), moderate: 55 (62%), mild: 5 (6%). Mean parity 2.1 (SD = 1.3). Number using medication: 44 (50%).	✓											↑ in incontinence episodes in the long term with a mean increase of 2.65 episodes for week
7	Lauti et al., 2008 [48]	RCT	bladder training + counseling	Women with predominant urge urinary incontinence experiencing at least monthly leakage and aged over 18 years. Mean age of the included women: 53.8 ± 14.8 (BRT), 63.9 ± 17.2 (DT), 47.6 ± 16.3 (COMBO).	✓	✓		Oxybutynin 2.5 mg/day			✓	bladder training and counseling + oxybutynin 2.5 mg/day	✓	✓	✓	=in incontinence episodes between groups
8	Pennisi et al., 1994 [53]	Quasi-experimental	physiotherapy	Women aged between 27 and 75 years old (mean age 51) with UI 121 women: 55 with SUI (20 treated with FKT, 35 with FKT + SEF), 20 with UUI (6 with FKT, 6 with FKT + Ditropan, 8 with FKT + SEF) and 46 with MUI (12 treated with FKT, 17 with FKT + Ditropan, 17 with FKT + SEF).	✓			Oxybutynin 5 mg/3 times per day			✓	physiotherapy + electric functional stimulation	✓			With physiotherapy, 83.3% of patients recovered, 26.7% improved. With the combination of physiotherapy and oxybutynin, 100% of patients recovered, and with the combination of physiotherapy and electric functional stimulation, 72.5% recovered and 37.5% improved their outcomes
9	Rasero and Mangani, 2005 [54]	Quasi-experimental	physiotherapy + electric functional stimulation	Women attending the pelvic floor rehabilitation clinic. No particular characteristics were required in order to access the rehabilitation program. Mean age of the included women: 57 years [range: 35-72].	✓											A total of 14.3% of women improved their outcomes, 14.3% recovered in the physiotherapy + electric functional stimulation group
10	Singh and Arya, 2011 [44]	Quasi-experimental	bladder rehabilitation program	A total of 12 motivated, non-demented (Mini Mental State Examination > 24) and ambulatory, community-dwelling subjects were taken for the study between ages of 55 and -70 years. Mean age of the included women: 63.75 ± 5.17 years. UI or predominant MUI persisting for at least three months with frequency of at least 2 or more episodes per week. Experience of involuntary loss of urine associated with strong desire to void.	✓											↓ in incontinence episodes (*p* = 0.000); statistical difference in pre–post tests of OABq and PIIQ with better scores (*p* = 0.000)
11	Smith, Boileau and Buan, 2000 [45]	Quasi-experimental	behavioral training + biofeedback	A total of 55 volunteer women with symptoms of urge, stress or mixed UI. The mean age was of 54 years [range 25–81]. Main characteristics of the participants: 37 were parous, 18 underwent a previous hysterectomy, 7 underwent a previous bladder surgery, 29 experienced both stress and urge symptoms and in 50%, symptoms lasted for more than 2 years. In total, 44 women completed the program.	✓	✓										↓ in incontinence episodes after the intervention and mean continence severity (*p* < 0.001)
12	Wadensten et al., 2021 [40]	RCT	behavioral training	A total of 123 women ≥ 18 years old with UUI or MUI and ≥2 leakages per week, access to a smartphone, and the ability to send and receive email. Mean age of the included women: 58 [range: 31–77] years. Treatment app (n = 60, 2 lost to follow-up) or the information app (control group, n = 63). Of these, 35 (28%) women had UUI, and 88 (72%) had MUI.	✓	✓		Information application		✓						↓ in incontinence episodes in the intervention group (*p* < 0.001)
13	Wu et al., 2021 [52]	RCT	behavioral and pelvic floor muscle training (B-PFMT)	A total of 647 postmenopausal women with: nocturia, urinary urgency and urinary frequency. Mean age: 62.9 ± 5.7	✓	✓		Informational DVD		✓						= in incontinence episodes between groups
14	Yoon, Song, and Ro, 2003 [46]	Quasi-experimental	bladder training	A total of 50 participants with urine loss or ≥ 14 voids every 48 h were recruited through public advertisements. All women were parous and aged 35–55.	✓			pelvic floor muscle training	✓							↓ in incontinence episodes in the bladder training group (*p* < 0.01)

**Table 4:** Other outcomes. “✓”indicates that the item has been included or mentioned in the study.

#	Authors and Year	Frequency of Incontinence Episodes	Incontinence Symptoms	Nocturia	Voided Volume	Patient’s Satisfaction	Quality of Life	Distress	Brain Activation
1	Burgio et al., 2008 [47]	✓				✓	✓	✓	
2	Burgio et al., 2010 [49]	✓							
3	Kafri et al., 2007 [42]	✓		✓			✓		
4	Ketai et al., 2021 [50]	✓							✓
5	Komesu et al., 2020 [51]	✓							
6	Lagro-Janssen and van Weel, 1998 [43]	✓							
7	Lauti et al., 2008 [48]	✓		✓			✓		
8	Pennisi et al., 1994 [53]		✓						
9	Rasero and Mangani, 2005 [54]		✓						
10	Singh and Arya, 2011 [44]	✓							
11	Smith, Boileau and Buan, 2000 [45]	✓							
12	Wadensten et al., 2021 [40]	✓	✓				✓		
13	Wu et al., 2021 [52]	✓		✓					
14	Yoon, Song, and Ro, 2003 [46]	✓		✓	✓				

Nocturia, defined as frequency per night by Kafri et al. (2007) [42], significantly improved in both rehabilitation group (behavioral training) and medication group (oxybutynin 5 mg/day) with a *p*-value of 0.001. Six months after the start of the intervention, the rehabilitation group obtained decreased episodes of nocturia compared to the pharmacological group (1.0 ± 0.9 vs. 1.9 ± 1.3) with a significant *t* test (*p* < 0.001) [42]. Lauti et al. (2008) [48] studied the number of voids per night in three different groups: bladder retraining (BRT), drug therapy with oxybutynin 2.5 mg/day (DT) and their combination (COMBO) [48]. They observed a reduction in nocturia episodes in all the groups at three months after the interventions, while in the following period until twelve months, the number of voids per night slightly increased. However, this study did not report the significant mean difference between any of the treatments for this outcome both at three months (COMBO vs. BRT: −0.6 [−1.9 to 0.8], *p* = 0.420; COMBO vs. DT: 0.3 [−1.1 to 1.7], *p* = 0.634; BRT vs. DT: 0.9 [−0.4 to 2.1], *p* = 0.160) and at twelve months after intervention (COMBO vs. BRT: −0.2 [−0.8 to 0.4], *p* = 0.579; COMBO vs. DT: −0.4 [−1.0 to 0.2], *p* = 0.209; BRT vs. DT: −0.2 [−0.8 to 0.3], *p* = 0.405) (Lauti et al., 2008) [48]. Nocturia was also examined by Wu et al. (2021) [52] in a study comparing a behavioral training and pelvic floor muscle training group with a control group, which saw an informational DVD. First group’s women complained of fewer nocturia episodes at three months (cOR = 2.1, 95% CI 1.6–2.9, *p* < 0.001), 12 months (cOR = 2.1, 95% CI 1.6–3.0, *p* < 0.001) and 24 months (cOR = 1.7, 95% CI 1.2–2.3, *p* = 0.005) after the intervention [52]. Another article investigated the effectiveness of bladder training compared to pelvic muscle exercises and did not find changes in nocturia during the eight-week posttreatment in the behavioral training group, while the outcome improved significantly in the bladder training group (repeated-measures ANOVA, *p* < 0.01) [46].

**Table 5:** Adverse events related to the provided interventions. “✓” indicates that the item has been included or mentioned in the study.

#	Authors and Year	Adverse Events	Non-Pharmacological Intervention	Pharmacological Intervention	Worst in	Description
1	Burgio et al., 2008 [47]	YES	✓	✓	not reported	blurred vision, syncope, night sweats, stomach cramping, weakness, small bowel obstruction, allergic reaction (pruritus and rash) and tachycardia (in the combination therapy group)
2	Burgio et al., 2010 [49]	NOT REPORTED				
3	Kafri et al., 2007 [42]	YES	✓	✓	pharmacological	dry mouth and fatigue
4	Ketai et al., 2021 [50]	NOT REPORTED				
5	Komesu et al., 2020 [51]	YES	✓	✓	pharmacological	constipation, dyspepsia, dry eyes, dry mouth, voiding difficulties, urinary tract infection, falls, headache, back pain (in the pharmacological group), urinary tract infection, falls, headache, back pain (in the hypnotherapy group)
6	Lagro-Janssen and van Weel, 1998 [43]	NOT REPORTED				
7	Lauti et al., 2008 [48]	YES	✓	✓	pharmacological	dry mouth, headache, dizziness–vertigo, constipation, fatigue
8	Pennisi et al., 1994 [53]	NOT REPORTED				
9	Rasero and Mangani, 2005 [54]	NO				
10	Singh and Arya, 2011 [44]	NOT REPORTED				
11	Smith, Boileau and Buan, 2000 [45]	NOT REPORTED				
12	Wadensten et al., 2021 [40]	YES	✓		behavioral training	inguinal hernia, altered incontinence symptoms
13	Wu et al., 2021 [52]	NOT REPORTED				
14	Yoon, Song, and Ro, 2003 [46]	NOT REPORTED				

## Data Availability

The data presented in this study are available on request from the corresponding author.

## Supplementary Materials

The following supporting information can be downloaded at: https://www.mdpi.com/article/10.3390/nursrep14010015/s1, Table S1: Search Strategies.

## Public Involvement Statement

No public involvement in any aspect of this research.

## Guidelines and Standards Statement

This manuscript was drafted against the Joanna Briggs Institute (JBI) manual for systematic reviews of effectiveness evidence.
