# Supplementary material for: Non-Pharmacological Management of Urge Urinary Incontinence in Women between 40 and 65 Years Old: A Systematic Review
Source: Nurs Rep. 2024 Jan 9;14(1):174–96. doi: 10.3390/nursrep14010015 (PMC10801617; doi:10.3390/nursrep14010015)
Supplement: Supplementary file 1 [file nursrep-14-00015-s001.zip › nursrep-2708642-supplementary.pdf]

Table S1. Electronic databases searches.

**Electronic database searches**					
**Date of search**	**Electronic Database**	**Search Strategies**	**Filters**	**Number of studies retrieved**	**Years**
17 ott. 2022	**MEDLINE via PubMed**	(("nonpharmacologic treatment*"[Title/Abstract] OR "nonpharmacologic intervention*"[Title/Abstract] OR "nonpharmacologic approach*"[Title/Abstract] OR "Behavior Therapy"[MeSH Terms] OR "behaviour therap*"[Title/Abstract] OR "behavioural therap*"[Title/Abstract] OR "Mindfulness"[MeSH Terms] OR "Mindfulness"[Title/Abstract]) AND ("urinary incontinence, urge"[MeSH Terms] OR "urge incontinen*"[Title/Abstract] OR "urge urinary incontinen*"[Title/Abstract] OR "urinary urgency"[Title/Abstract] OR "UUI"[Title/Abstract]) AND ("rehabilitation" OR "Rehabilitation"[MeSH Terms]) AND ("female"[MeSH Terms] AND ("english"[Language] OR "italian"[Language]))) AND (female[Filter])	Language:<br>- English<br>- Italian<br><br>Sex: Female	56	1985-2019
	**PsycInfo [EBSCO]**	(("nonpharmacologic treatment*" OR "nonpharmacologic intervention*" OR "nonpharmacologic approach*" OR "behaviour therap*" OR "behavioural therap*" OR (MM "Rehabilitation") OR "Mindfulness" OR (MM "Mindfulness-Based Interventions")) AND ((MM "Urinary Incontinence") OR "urge incontinen*" OR "urge urinary incontinen*" OR "urinary urgency" OR "UUI") AND ("Rehabilitation" OR (MM "Rehabilitation"))	Language:<br>- English<br>- Italian<br><br>Sex: Female	7	1997-2019
	**Scopus [Elsevier]**	(TITLE-ABS-KEY (("nonpharmacologic treatment*" OR "nonpharmacologic intervention*" OR "nonpharmacologic approach*" OR "Behavior Therapy" OR "behaviour therap*" OR "behavioural therap*" OR "Mindfulness"))) AND (TITLE-ABS-KEY (("urge incontinen*" OR "urge urinary incontinen*" OR "urinary urgency" OR "UUI"))) AND (TITLE-ABS-KEY (rehabilitation)) AND (LIMIT-TO (LANGUAGE , "English") OR LIMIT-TO (LANGUAGE , "Italian")) AND (LIMIT-TO (EXACTKEYWORD , "Female"))	Language:<br>- English<br>- Italian<br><br>Sex: Female	7	1997-2021
	**CINAHL [EBSCO]**	(("nonpharmacologic* treatment*" OR "nonpharmacologic* intervention*" OR "nonpharmacologic* approach" OR "behaviour therapy" OR "behavioural	Language:<br>- English	168	1972-2022

		therapy" OR (MH "Behavior Therapy+")OR (MH "Mindfulness+") OR (MH "Mind Body Techniques+") OR "mindfulness" OR "behaviour therapy" OR "behavioural therapy") AND ((MH "Urge Incontinence") OR (MH "Urge Urinary Incontinence (Saba CCC)") OR (MH "Urge Incontinence (NANDA)" OR "urge incontinen*" OR "urge urinary incontinen*" OR "UUI" OR "urinary incontinen* rehabilitation") AND ((MM "Rehabilitation Patients") OR "rehabilitation")	- Italian<br><br>Sex: Female		
	**Embase [Elsevier]**	('nonpharmacologic treatment*' OR 'nonpharmacologic intervention*' OR 'nonpharmacologic approach*' OR 'behavior therapy'/exp OR 'behavior therapy' OR 'behaviour therap*' OR 'behavioural therap*' OR 'mindfulness'/exp OR 'mindfulness') AND ('urinary incontinence, urge'/exp OR 'urinary incontinence, urge' OR 'urge incontinen*' OR 'urge urinary incontinen*' OR 'urinary urgency'/exp OR 'urinary urgency' OR 'uui') AND ('rehabilitation'/exp OR 'rehabilitation')	LANGUAGE:<br>- English<br>- Italian<br><br>Sex: Female<br>Article<br>Review	43	1985-2021
	**Web of Science**	ALL=(('nonpharmacologic treatment*' OR 'nonpharmacologic intervention*' OR 'nonpharmacologic approach*' OR 'behavior therapy' OR 'behaviour therap*' OR 'behavioural therap*' OR 'mindfulness') AND ('urinary incontinence, urge' OR 'urge incontinen*' OR 'urge urinary incontinen*' OR 'urinary urgency' OR 'uui') AND ('rehabilitation'))	LANGUAGE:<br>- English, SEX:<br>Women OR<br>Female	7	2013-2020
